# Bile acids targeted metabolomics and medication classification data in the ADNI1 and ADNIGO/2 cohorts

**DOI:** 10.1038/s41597-019-0181-8

**Published:** 2019-10-17

**Authors:** Lisa St. John-Williams, Siamak Mahmoudiandehkordi, Matthias Arnold, Tyler Massaro, Colette Blach, Gabi Kastenmüller, Gregory Louie, Alexandra Kueider-Paisley, Xianlin Han, Rebecca Baillie, Alison A. Motsinger-Reif, Daniel Rotroff, Kwangsik Nho, Andrew J. Saykin, Shannon L. Risacher, Therese Koal, M. Arthur Moseley, Jessica D. Tenenbaum, J. Will Thompson, Rima Kaddurah-Daouk, Alzheimer’s Disease Neuroimaging Initiative, Alzheimer’s Disease Metabolomics Consortium

**Affiliations:** 1 Proteomics and Metabolomics Shared Resource, Center for Genomics and Computational Biology, Duke University, Durham, NC USA; 2 Department of Psychiatry & Behavioral Sciences, Duke University, Durham, NC USA; 3 Institute of Bioinformatics and Systems Biology, Helmholtz Zentrum München, German Research Center for Environmental Health, Neuherberg, Germany; 4 Duke Clinical Research Institute, Duke University, Durham, NC USA; 5 Duke Molecular Physiology Institute, Duke University, Durham, NC USA; 6 German Center for Diabetes Research, Neuherberg, Germany; 7 University of Texas Health Science Center at San Antonio, San Antonio, TX USA; 8 Rosa & Co LLC, San Carlos, CA USA; 9 Bioinformatics Research Center, Department of Statistics, North Carolina State University, Raleigh, NC USA; 10 Department of Quantitative Health Sciences, Cleveland Clinic, Cleveland, OH USA; 11 Department of Radiology and Imaging Sciences and the Indiana Alzheimer Disease Center, Indiana University School of Medicine, Indianapolis, IN USA; 12 BIOCRATES Life Sciences AG, Innsbruck, Austria; 13 Department of Biostatistics and Bioinformatics, Duke University, Durham, NC USA; 14 Duke Institute for Brain Sciences, Duke University, Durham, NC USA

**Keywords:** Bioinformatics, Metabolomics, Alzheimer's disease

## Abstract

Alzheimer’s disease (AD) is the most common cause of dementia. The mechanism of disease development and progression is not well understood, but increasing evidence suggests multifactorial etiology, with a number of genetic, environmental, and aging-related factors. There is a growing body of evidence that metabolic defects may contribute to this complex disease. To interrogate the relationship between system level metabolites and disease susceptibility and progression, the AD Metabolomics Consortium (ADMC) in partnership with AD Neuroimaging Initiative (ADNI) is creating a comprehensive biochemical database for patients in the ADNI1 cohort. We used the Biocrates Bile Acids platform to evaluate the association of metabolic levels with disease risk and progression. We detail the quantitative metabolomics data generated on the baseline samples from ADNI1 and ADNIGO/2 (370 cognitively normal, 887 mild cognitive impairment, and 305 AD). Similar to our previous reports on ADNI1, we present the tools for data quality control and initial analysis. This data descriptor represents the third in a series of comprehensive metabolomics datasets from the ADMC on the ADNI.

## Abstract (summary)

**Table:** 

Measurement(s)	bile acid • Medication
Technology Type(s)	ultra high performance liquid chromatography with mass spectrometer • Resource Informatics
Factor Type(s)	age • biological sex • cognitive state
Sample Characteristic - Organism	Homo sapiens

Machine-accessible metadata file describing the reported data: 10.6084/m9.figshare.9724652

## Background and Summary

With the dramatic increase of older adults around the world, Alzheimer’s disease (AD) has become a major public health challenge^1^. AD is the leading cause of dementia, and is clinically defined by an insidious onset and a progressive loss of memory and other cognitive functions that effects a person’s ability to function in daily activities^2^. AD is a complex and progressive disorder: the cognitive and functional decline is preceded by a pre-clinical phase known as mild cognitive impairment (MCI). MCI is a complex syndrome characterized by memory failures that may be considered as an intermediate stage in the development of AD and is distinct from normal aging^3^.

The etiology of AD is highly complex and multifactorial. Accumulating evidence highlights numerous biochemical perturbations that are suggested to play a role in AD. These include the characteristic deposition of β-amyloid plaques (Aβ), hyperphosphorylation of tau protein, oxidative stress, inflammation, abnormal metal homeostasis, as well as disruption in energetic and neurotransmitter pathways, among others^4–9^. AD has traditionally been considered primarily a neurodegenerative disorder of the central nervous system, but there is increasing evidence that the pathological processes associated with disease may also manifest in the peripheral system^10–12^.

Our limited understanding of the etiology is reflected in the limited options for therapeutic treatments^13^. There are currently a limited number of treatments available, and they have only modest effects^14^. A recent review of drug development in AD discusses these issues in detail^15^. Improved mechanistic understanding of disease onset and progression is central to more efficient AD drug development and will lead to improved therapeutic approaches and targets.

To better understand this complex etiology, the application of metabolomics for AD research has the potential to monitor molecular alterations associated with disease pathogenesis and progression, as well as to discover candidate diagnostic biomarkers. The rapidly emerging field of metabolomics combines strategies to identify and quantify cellular metabolites using sophisticated analytical technologies with the application of statistical and multi-variant methods for information extraction. Current technologies allow for the high throughput collection of a large number of metabolites^16,17^. Initial studies have demonstrated the potential of metabolomics in AD, demonstrating that metabolomics markers may serve as biomarkers for the disease, and help unravel the complex biochemical pathways involved in the disease^4,5,18–23^.

These successes motivate the collection of metabolomics data in large, well-powered cohorts. The Alzheimer’s Disease Neuroimaging Initiative (ADNI) is a public-private partnership that has established a landmark longitudinal cohort to increase the rate of scientific discovery in AD. Data collection for the ADNI cohort is comprehensive. Across thousands of subjects, ADNI researchers collect MRI and PET images, genetics, cognitive tests, CSF and blood biomarkers, etc.^24,25^. Details of the ADNI efforts can be found at www.adni-info.org. The Alzheimer’s Disease Metabolomics Consortium (ADMC) is working with ADNI to add metabolomics data to the vast collection of data collected for this cohort. The data collected through the ADMC will provide a resource to interrogate global metabolomics changes within the ADNI cohort, to enhance the systems level data available. A total of eight targeted and non-targeted metabolomics platforms are being used by the ADMC, with the second of these described in the current manuscript.

Herein, we describe the use of the Biocrates Bile Acids kit to profile baseline serum samples from the ADNI1 and ADNIGO/2 cohorts. We also apply the medication mapping approach performed previously on ADNI1 to the ADNIGO2 cohort^23^. These data are intended to aid in the discovery of metabolic features associated with disease risk, progression, or other clinically and biologically relevant outcomes. We describe both the data collection and tools and resources for data processing, quality control, and analysis.

## Methods

### Alzheimer’s disease neuroimaging initiative (ADNI) cohort

ADNI data was obtained through the University of Southern California’s Laboratory of Neuroimaging (LONI) data repository (http://adni.loni.ucla.edu/), where data and results have been made accessible through the AMP-AD Knowledge Portal (https://ampadportal.org). The AMP-AD Knowledge Portal is the distribution site for data, analysis results, analytical methodology and research tools generated by the AMP-AD Target Discovery and Preclinical Validation Consortium and multiple Consortia and research programs supported by the National Institute on Aging. Institutional Review Board approval and written informed consent was obtained at each of the participating institutions. Data obtained included: demographic information, clinical assessment data, clinical diagnosis, etc. Detailed information on the ADNI cohort is described in Petersen *et al*. 2010 and at http://www.adni-info.org/ ^24^. ADNI data collection is ongoing, and variables are continually updated through their LONI resource. Data presented here are from January 2016, and represent a snapshot of the clinical data for analytical reproducibility. A summary of key demographic and clinical variables are summarized in Table 1 for ADNI 1 and Table 2 for ADNIGO/2.

**Table 1:** Demographics and clinical data of studied ADNI subjects at baseline.

	CN (n = 193)	LMCI (n = 357)	AD (n = 172)	p-value
Age, year M (SD)	75.60 (4.90)	74.71 (7.44)	75.09 (7.35)	0.84
Sex, Female % (n)	50% (96)	35% (126)	49% (84)	<0.001
APOE ε4, % (n)	28% (54)	53% (189)	67% (115)	<0.001
MMSE, M (SD)	29.17 (0.96)	26.96 (1.81)	23.44 (2.00)	<0.0001
ADAS-Cog13, M (SD)	9.33 (4.16)	18.61 (6.32)	28.57 (7.66)	<0.0001

Abbreviations: AD-Alzheimer’s Disease; ADAS-Cog13: Alzheimer’s Disease Assessment Scale-Cognitive subscale; CN-cognitively normal, MMSE-Mini-Mental State Exam, LMCI-late mild cognitive impairment.

**Table 2:** Demographics and clinical data of studied ADNIGO/2 subjects at baseline.

	CN (n = 177)	SMC (n = 98)	EMC (n = 284)	LMCI (n = 148)	AD (n = 133)	p-value
Age (years)	73.46 (6.29)	72.18 (5.63)	71.12 (7.51)	72.12 (7.64)	74.21 (8.33)	<0.001
Sex, Female % (n)	53% (94)	57% (56)	46% (130)	48% (71)	41% (55)	0.09
APOE ε4, % (n)	28% (50)	33% (32)	43% (121)	57% (84)	65% (87)	<0.001
MMSE, M (SD)	29.03 (1.27)	29.00 (1.20)	28.32 (1.60)	27.64 (1.79)	23.06 (2.06)	<0.001
ADAS-Cog13, M (SD)	9.05 (4.48)	8.78 (4.12)	12.64 (5.40)	18.80 (7.31)	31.10 (8.67)	<0.001

Abbreviations: AD-Alzheimer’s disease; ADAS-Cog13: Alzheimer’s Disease Assessment Scale-Cognitive subscale; CN-cognitively normal; EMCI: early mild cognitive impairment; MMSE-Mini-Mental State Exam; LMCI-late MCI; SMC- subjective memory complaints.

### Serum collection and sample management

Samples were collected at the baseline study visit. Blood samples were collected in the morning, after overnight fasting (except where explicitly annotated). Standard operating procedures are detailed at (www.adni-info.org). Briefly, duplicate blood samples were collected in two bar-coded 10 mL red-top plastic Vacutainer blood tubes, allowed to clot for 30 minutes, and then centrifuged at 3000 rpm (1500 rcf) for 15 minutes. The serum was then transferred into a bar-coded 13 mL polypropylene transfer tube, capped and frozen in dry ice. Frozen samples were overnighted to the ADNI biomarker core laboratory at the University of Pennsylvania Medical Center. Samples were thawed and aliquoted to 0.5 mL samples, then once more for individual laboratory analyses. A 20 µL sample aliquot was delivered to the Duke Proteomics and Metabolomics Shared Resource (Durham, NC) for analysis with the Bile Acids platform, as detailed below.

### Metabolomics analysis using the biocrates bile acids kit

#### Sample preparation

**Fig. 1:**
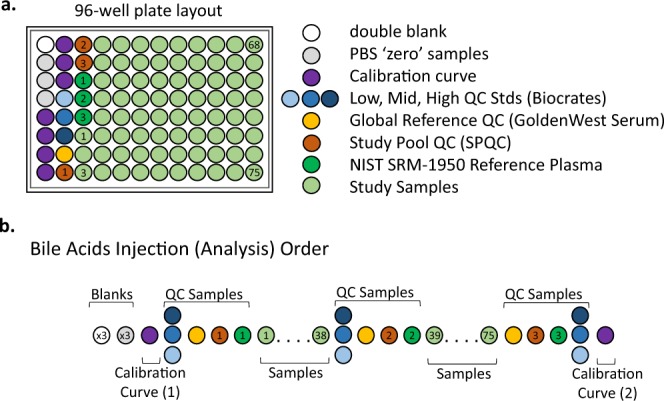
Plate layout for quantitative bile acids analysis in ADNI cohorts. (**a**) 96-well plate layout used for sample preparation and data collection for the bile acids metabolomics analysis by LC-MS/MS. Each of the plates analyzed in the study used the same lot of calibrators, Biocrates QCs, study pool QC (SPQC), GoldenWest Serum and NIST SRM-1950 plasma. (**b**) Analysis order for each plate showing how the calibration curve and QC samples bracket the actual sample analyses, following FDA guidance for regulated bioanalysis, in order to decrease the likelihood of intraplate bias.

Samples were prepared and analyzed in the Duke Proteomics and Metabolomics Shared Resource using the Biocrates® Bile Acids Kit (Biocrates Life Sciences AG, Innsbruck, Austria) in accordance with the user manual. In brief, 10 µL of the supplied internal standard solution were added to each well (except for the zero sample) on a filterspot of the 96-well extraction plate. After drying under a gentle stream of nitrogen 10 µL of each serum sample, quality control (QC) samples, blank, zero sample, or calibration standard were added to the appropriate wells (Fig. 1). The plate was then dried under a gentle stream of nitrogen. Sample extract elution was performed with methanol. Sample extracts were diluted with water for UPLC-MS/MS.

#### Quality control samples

The analysis of the samples using the Biocrates® Bile Acids Kit was performed using four specific sets of quality controls. First, low/mid/high level QC samples provided by Biocrates Life Sciences AG were prepared and analyzed on each plate as recommended by the manufacturer. These QC samples were used for a technical validation of each kit plate. Second, to allow appropriate inter-plate abundance scaling based specifically on this cohort of samples, we generated a Study Pool QC (SPQC) by combining approximately 10 µL from the first 75 samples for analysis. This sample was frozen in aliquots of 45 uL then prepared and analyzed three times on each plate. Third, the study utilized 18 blinded analytical duplicates and 15 blinded analytical triplicates for ADNI 1 and ADNIGO/2, respectively. These replicates, obtained from the same serum draw, were scattered throughout the study in a manner blinded to the investigators until data was sent to the ADNI informatics core for unblinding. The commonly used reference materials NIST SRM-1950 plasma (n = 3 per plate) and GoldenWest serum pool (n = 1 per plate) were also analyzed on each plate to allow cross-comparison against other sample cohorts in the future.

Figure 1a shows the preparation layout for the 96-well plates as utilized in this study. In total, eleven plates were prepared in order to analyze serum samples for ADNI1 (Oct–Nov 2015) along with the blinded replicates. The same approach was used in the analysis of ADNIGO/2 cohort (Oct–Nov 2016) plus blinded replicates. The blank, zero sample, calibration standards, and Low/Mid/High QC samples provided with the kit were arranged as recommended by Biocrates. In order to improve the ability to compare results with other metabolomics studies and reduce plate-to-plate batch effects, seven additional wells were used for the additional QC samples as described above: three wells for the study pool QC (SPQC), one well for the GoldenWest Pooled Serum Standard, and three wells for the NIST SRM-1950 Standard Reference Plasma. The remaining 75 wells were used for cohort samples. The analysis order of each plate is summarized in Fig. 1b. The order was arranged to maximize quantitative accuracy and precision within a plate, and limit the potential for batch effects. The analysis order included running the standard curve twice, once at the beginning and end of the samples. The Biocrates QCs and GoldenWest Serum QC were prepared once but injected in technical triplicate, once before, in the middle (after 38 samples), and at the end of the sample set. The SPQC samples (n = 3) were each analyzed once, with one analysis before, in the middle, and one after all samples on the plate. The NIST SRM-1950 plasma (n = 3) were also analyzed once each at the beginning, middle, and end of the cohort samples. Bracketing the standard curves and nesting the analytical samples between the QCs offers the best chance of observing any system drift and assuring optimal instrument performance across the sample set.

#### Quantitative UPLC-MS/MS analysis

Mass spectrometry analysis was performed based on Standard Operating Procedure (SOP #8111) provided by Biocrates for the Bile Acids kit. Chromatographic separation of the analytes was performed using an ACQUITY UPLC System (Waters Corporation) using a proprietary reverse-phase UPLC and guard column provided by Biocrates then quantified by calibration curve using a linear regression with 1/x^2^ weighting. Samples were introduced directly into a Xevo TQ-S mass spectrometer (Waters Corporation) using negative electrospray ionization operating in the Multiple Reaction Monitoring (MRM) mode. MRM and pseudo-MRM transitions (compound-specific ions) for each analyte and internal standard were collected over a scheduled retention time window using tune files and acquisition methods provided in the Biocrates® Bile Acids kit. The UPLC data were imported into TargetLynx (Waters Corporation) for peak integration, calibration, and concentration calculations. The UPLC data from TargetLynx were analyzed using Biocrates’ Met*IDQ* v5.4.8 software. The kit data are reported in detail in the Supplemental Information on LONI, along with a color-coded key denoting samples that were below the limit of detection (<LOD) or below the lowest calibration standard (<LLOQ). The data generated for the study samples and SPQC samples can be downloaded using the appropriate links contained in Online-only Table 1.

### Data processing

**Fig. 2:**
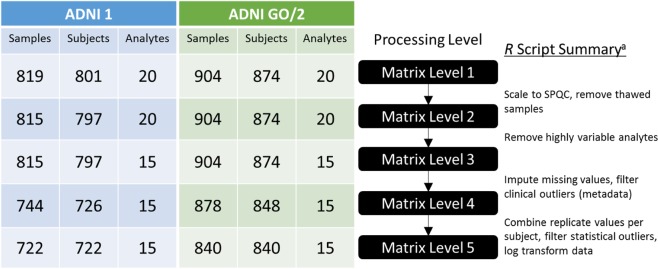
Workflow description for data curation and scaling of the bile acids data. The use of Levels breaks the workflow into discrete steps which can be applied to multiple metabolomics data types, and will be consistent across the eight metabolite datasets collected for ADNI. Filtering for ADNI 1 is shown on left, and ADNI GO/2 is shown on the right. The workflow executed in R is described on the right. *Subjects flagged for exclusion in Level 4 are not physically excluded from the table until Level 5.

R v3.2.4 (www.r-project.org) statistical software was used for data analysis. R scripts for data processing are available at links provided in Online-only Table 1. The workflow for processing data from the raw set of concentration values for each subject in each cohort to something that is prepared for statistical analysis includes a series of important steps as previously described for the AbsoluteIDQ p180 platform dataset in ADNI 1^23^. Figure 2 provides the graphical representation of the important steps in this process for the Bile Acids datasets for ADNI 1 and ADNI 2/GO. We briefly describe the overall data processing with different levels of the data, as different stages of quality control and processing below, but detailed descriptions of each step can be found in prior publications^23^ or in the supplemental material described in Online-only Table 1. The input to this pipeline, formerly called “Level 0”, is the *.xlsx or *.csv format measured concentrations exported from the Biocrates software, MetIDQ.

The first step of QC was to exclude four samples that were inadvertently included in the ADNI 1 cohort, and to scale the quantitative value of each metabolite across plates and cohorts using the pooled NIST samples that were analyzed three times in ADNI1 and twice in ADNIGO/2 on each plate. Scaling was done by dividing the global average of each metabolite level by its average within the plate. These batch effect adjusted values are included in the Intermediate Data Level 2. Overall, this scaling factor was small (typically less than 10%), as can be seen by the raw reported NIST SRM-1950 values reported in Online-only Table 2.

The next step of QC involved filtering based on quality metrics. We routinely applied filter criteria to each of the metabolites (based on the blinded ADNI 1 duplicates or ADNI 2/GO triplicates) to allow only the most robust analytes to be included in downstream analysis. Separately for each cohort, we used a coefficient of variation (CV) <30% across plates to filter out metabolites with limited variation and therefore statistical power for analysis. Next, we used an intraclass correlation coefficient (ICC) between the values for the blinded duplicate (or triplicate) analyes >0.6. Finally, analytes with >40% of measurements below the lower limit of detection (<LOD) were filtered out. This filtered data represents the Level 3 data matrix. This filtering reduced the total number of analytes reported from 20 analytes (Level 2) to 15 analytes (Level 3). The filter QC results are presented in detail in the Supplementary Table 1.

The next step in data processing performs missing value replacement, by imputing any values reported as reported as ‘<LOD’ were using LOD/2 value for each specific analyte. Additionally, we screened for outliers for removal prior to analysis. In ADNI 1, there were a total of 71 samples identified as outliers based on the following criteria: 69 samples identified as non-fasting, 2 samples lacking corresponding body mass index (BMI) values, and 1 for which no baseline medication record was reported. The resulting Intermediate Data – Level 4 contained n = 744 samples (726 subjects) and n = 15 analytes. In ADNI GO/2, there were a total of 26 non-fasting samples that resulted in the Intermediate Data – Level 4 contained n = 878 samples (848 subjects) and n = 15 analytes.

The final step of data processing (Level 5) prior to analysis achieved the following goals. First, duplicate/triplicates measures for the blinded duplicates were averaged to give singular values for each analyte for each sample. An additional screen for outliers was performed from a statistical perspective using Principal Components Analysis (PCA). Principal components that explained >90% cumulative variance were selected, and any subjects located more than 7 SD from the mean were filtered out as outliers. This identified 4 and 6 additional samples, respectively, that were excluded from the final data matrices for ADNI 1 and ADNI 2/GO. Finally, all metabolites were log2 transformed. The final, analysis-ready data matrix (Fig. 2, Matrix Level 5) contained 722 and 840 subjects in ADNI 1 and ADNI GO/2, respectively, and 15 analytes.

Because this processing represents an initial analysis, relatively conservative quality control was performed. A major motivation of the different levels of data within the workflow was to provide data at different levels of processing for further evaluation, including analyses targeting different hypotheses and using different assumptions than undertaken in the initial analysis. Full transparency is emphasized, as data utilized or generated in each step of the pipeline is available in Online Table 1^26–40^.

### Collection and curation of medication data

As with any other observational cohort, there are a number of challenges with confounding in the study design. We previously reported an informatics framework which utilizes the free text medication information available on LONI as well as the National Library of Medicine’s (NLM) RxNorm API (application programming interface) to match drug names to standardized drug concept identifiers, thus creating a common set of Boolean flags for each patient, annotating whether or not they were taking a drug in a specific class^23^. These binary flags make it much more straightforward to include medications in statistical analysis to assess potential confounding in subsequent association analyses. The same approach was utilized in this work, applied now to both ADNI1 and ADNIGO/2 cohorts. The code for this processing pipeline, along with documentation and API configuration files is available in Synapse: 10.7303/syn7477310 ^41^. The final table of Boolean variables for each drug class at the baseline visit for ADNI1 is available at 10.7303/syn7440367.1 ^42^, and ADNIGO/2 is available 10.7303/syn12179110.1 ^43^.

### Data Records

Online-only Table 1 details the location of each of the files generated in this study (called Data in the “Type” column) or utilized in this study, which are is hosted on the AMP-AD Knowledge Portal (https://ampadportal.org). The Online-only Table 1 includes links to R scripts for data processing, processing pipeline for medication data along with documentation and API configuration files, as well as links to data from both ADNI 1 and ADNI GO/2 for Bile Acids in various stages^26–45^.

Data use restrictions prohibit the distribution of any ADNI clinical or demographic data outside of LONI, so the data files to be input into the processing pipeline are hosted there. Researchers can apply for access to the ADNI data at https://ida.loni.usc.edu/collaboration/access/appLicense.jsp. Online-only Table 1 details the location of each of the files needed to reproduce the data presented here. The data must be downloaded from LONI (clinical data and medication data), and placed in the same directory as the scripts provided. There are points in some of the scripts where manual intervention is necessary as detailed in readme files that accompany the scripts.

## Technical Validation

The Bile Acids kit from Biocrates was validated according to European Medicine Agency Guideline on bioanalytical method validation. Additionally, the methodologies utilized in this study performed within the Duke Proteomics and Metabolomics Shared Resource include bracketing calibration curves and quality control samples throughout the run list of each plate, consistent with the FDA Guidance on Bioanalytical Method Validation. Additionally, the measurement of 17 human and 20 total bile acids in plasma using this kit was recently shown to have bias <30% and an average %CV <15% across 12 laboratories in an international ring trial^46^. Each kit includes an automated technical validation based on Quality Control samples provided by the vendor (Biocrates AG), which are plasma samples spiked at three different levels. The low, mid, and high QC samples serve to verify the analytical performance of each kit once the data is imported into the MetIDQ software package (Biocrates AG). A specific standard (calibrator) was considered valid, and included in sample quantification, when the backcalculated quantity (bias) was within 20% of the expected value at the lower limit of quantification (LLOQ) and within 15% at all points above the LLOQ. As a technical note, specific compounds within this kit can have high MS background (for example LCA), and it is important to utilize the test mix provided by Biocrates as a system suitability test. The most common reason for high background prior to sample analysis was found to be contaminants in the formic acid or methanol used to make the mobile phase, which was alleviated by utilizing formic acid only from glass ampules and fresh bottles of LC-MS grade methanol.

## Usage Notes

The general ADNI data use agreements and access policies apply to investigators using the metabolomics data. For details on how to apply for access and rules of usage, please see: http://adni.loni.usc.edu/data-samples/access-data/.

It is also important to note that users must apply for access and accounts for both ADNI and Synapse separately.

## Supplementary information

Supplemental Table 1

## Data Availability

We are highly committed to sharing all resources used to produce this data and analysis. Primary distribution of the scripts used in analysis is available through Sage Bionetworks’ Synapse platform through the AMP-AD Knowledge Portal (https://ampadportal.org), with links to the data from both ADNI 1 and ADNI GO/2 for Bile Acids in various stages, as well as the R scripts used to process the data, available at the links shown in Online-only Table 1.

## Online-only Tables

**Online-only Table 1:** Names, types, descriptions, and locations of primary data and additional files included in this data set.

File Name	Description	Type	Location	URL
Top Level ADNI1 Project Page^26^	Synapse Portal page for AMP-AD ADNI1 project	Portal	Synapse	http://dx.doi.org/10.7303/syn5592519
Top Level ADNI2-GO Page^27^	Synapse Portal page for AMP-AD ADNI2-GO project	Portal	Synapse	http://dx.doi.org/10.7303/syn9705278
**Primary Metabolomics Files**				
ADMCBA.csv^28^	ADNI1 Bile Acid Data, concentrations	Data	LONI	http://dx.doi.org/10.7303/syn12036817.1
ADMCBA_DICT.csv^29^	ADNI1 Bile Acids Data dictionary	Data Dict	LONI	http://dx.doi.org/10.7303/syn12036821.1
ADNI_ADMC_Bile_Acids_Method_Description_20160121.pdf^30^	ADNI1 Bile Acid Methods Description	Methods	LONI	http://dx.doi.org/10.7303/syn12036820.1
ADNI_ADMCM2OVEBA.csv^31^	ADNI2-GO Bile Acid Data, concentrations	Data	LONI	http://dx.doi.org/10.7303/syn9779093.1
ADNI_ADMCM2OVEBA_DICT.csv^32^	ADNI2-GO Bile Acids Data dictionary	Data Dict	LONI	http://dx.doi.org/10.7303/syn9779094.1
ADMC_ADNIGO2_Bile_Acids_Method_Description.pdf^33^	ADNI2-GO Bile Acid Methods Description	Methods	Synapse	http://dx.doi.org/10.7303/syn9779078.1
**Supplemental Metabolomics Files**				
ADNI Bile Acids _LEVEL0_to_LEVEL1.R etc.^34^	Data processing R scripts	Scripts	Synapse	http://dx.doi.org/10.7303/syn12036815
4226_Bile_Acids_NIST_and_QC_Data.xlsx^35^	ADNI1 NIST and QC values	Supp data	Synapse	http://dx.doi.org/10.7303/syn9779088.1
ADNI1 BA LOD values.csv^36^	ADNI1 LOD values	Supp data	Synapse	http://dx.doi.org/10.7303/syn12046012.1
4543_Bile_Acids_QC_and_NIST_Data.xlsx^37^	ADNI2-GO NIST and QC	Supp data	Synapse	http://dx.doi.org/10.7303/syn9779088.1
BILEACIDSLODvalues.csv^38^	ADNI2-GO LOD values	Supp data	Synapse	http://dx.doi.org/10.7303/syn9779079.1
BileAcidRatios_revised_2017_12_07.csv^39^	ADNI1 Bile Acid Ratios	Supp data	Synapse	http://dx.doi.org/10.7303/syn12046208.1
Fasting Status-ADNI1.txt^40^	ADNI Fasting Status	Supp data	Synapse	http://dx.doi.org/10.7303/syn12046023.1
**Clinical and Medication files**				
ADNI_All_Clinical_Data_16May2016.csv^44^	Clinical variables (a subset of ADNI’s complete list) snapshot from May, 2016	Data	LONI	http://dx.doi.org/10.7303/syn7477271.1
RECCMEDS.csv^45^	Original medication data- all cohorts, all timepoints. NOT versioned.	Data	LONI	http://dx.doi.org/10.7303/syn7829508.1
Medication mapping pipeline files^43^	Scripts and config files for medication concept mapping and classification	Scripts	Synapse	http://dx.doi.org/10.7303/syn7477310
ADMCADNI1SCPATIENTDRUGCLASSES.csv^41^	Results file mapping participants to classes of drugs taken at baseline for ADNI1	Supp data	LONI	http://dx.doi.org/10.7303/syn7440367.1
Patient drug classes for ADNIGO2^42^	Results file mapping participants to classes of drugs taken at baseline for ADNIGO2	Supp data	LONI	http://dx.doi.org/10.7303/syn12179110.1

**Online-only Table 2:** Bile Acid analytes measured.

Bile Acid	Abbreviation	LOD (µM)	Lowest CS (µM)	Highest CS (µM)	Average NIST SRM 1950 (µM)	Std Dev NIST SRM 1950 (µM)	Average Study Pool QC(µM)	Std Dev Study Pool QC (µM)
Cholic acid	CA	0.004	0.03	75	0.127	0.006	0.290	0.020
Chenodeoxycholic acid	CDCA	0.005	0.02	30	0.302	0.016	0.406	0.020
Deoxycholic acid	DCA	0.006	0.02	10	0.385	0.016	0.737	0.024
Glycocholic acid	GCA	0.003	0.03	75	0.271	0.012	0.224	0.011
Glycochenodeoxycholic acid	GCDCA	0.010	0.02	20	1.116	0.048	0.617	0.020
Glycodeoxycholic acid	GDCA	0.010	0.01	10	0.504	0.019	0.462	0.013
Glycolithocholic acid	GLCA	0.006	0.01	5	0.022	0.002	0.019	0.002
Glycolithocholic acid sulphate	GLCAS	0.028	NA	NA	0.463	0.030	0.528	0.040
Glycoursodeoxycholic acid	GUDCA	0.006	0.01	10	0.181	0.006	0.110	0.004
Hyodeoxycholic acid	HDCA	0.005	0.01	5	<LOD	NA	<LOD	NA
Lithocholic acid	LCA	0.002	0.01	5	0.007	0.011	0.021	0.015
Muricholic acid, alpha	MCA(a)	0.008	0.005	5	<LOD	NA	<LOD	NA
Muricholic acid, beta	MCA(b)	0.008	0.01	10	<LOD	NA	<LOD	NA
Muricholic acid, omega	MCA(o)	0.007	0.005	5	<LOD	NA	<LOD	NA
Taurocholic acid	TCA	0.008	0.02	50	0.023	0.005	0.048	0.006
Taurochenodeoxycholic acid	TCDCA	0.005	0.01	20	0.089	0.004	0.082	0.003
Taurodeoxycholic acid	TDCA	0.001	0.01	10	0.041	0.002	0.057	0.002
Taurolithocholic acid	TLCA	0.001	0.01	5	0.002	0.0009	0.003	0.0009
Taurolithocholic acid sulphate	TLCAS	0.040	NA	NA	0.076	0.018	0.099	0.030
Tauromuricholic acid (alpha + beta)	TMCA (a + b)	0.001	0.01	10	0.007	0.0011	0.007	0.001
Tauroursodeoxycholic acid	TUDCA	0.001	0.01	15	0.006	0.001	0.006	0.001
Ursodeoxycholic acid	UDCA	0.002	0.02	30	0.087	0.007	0.066	0.009

The table below shows the bile acid analytes measured in this kit, the defined lower limit of detection, calibration range (given as lowest calibration standard to highest calibration standard), the average and standard deviation for each measured value in the human plasma reference standard (NIST SRM-1950), and in the Study Pool QC.
