# Supplementary material for: Bile acids targeted metabolomics and medication classification data in the ADNI1 and ADNIGO/2 cohorts
Source: Sci Data. 2019 Oct 17;6:212. doi: 10.1038/s41597-019-0181-8 (PMC6797798; doi:10.1038/s41597-019-0181-8)
Supplement: Supplementary file 1 — Supplemental Table 1 [file 41597_2019_181_MOESM1_ESM.docx]

**Supplemental Table 1.** Processing using the workflow described in Figure 2, the table below reports the quality control metrics including fraction of missing data, coefficient of variation (CV), and intraclass correlation coefficient (ICC) for each analyte.

|  | **ADNI-1** | | | **ADNI-GO/2** | | |
| --- | --- | --- | --- | --- | --- | --- |
| **Analyte** | **Fraction missing** | **CV** | **ICC** | **Fraction missing** | **CV** | **ICC** |
| **CA** | **1.84%** | **0.08** | **0.99** | **0.44%** | **0.09** | **1.00** |
| **CDCA** | **7.48%** | **0.15** | **1.00** | **6.64%** | **0.13** | **1.00** |
| **DCA** | **1.23%** | **0.08** | **0.99** | **1.44%** | **0.07** | **0.98** |
| **GCA** | **0.12%** | **0.09** | **0.97** | **0.00%** | **0.06** | **0.99** |
| **GCDCA** | **0.00%** | **0.07** | **0.98** | **0.00%** | **0.06** | **0.99** |
| **GDCA** | **1.35%** | **0.06** | **0.99** | **1.77%** | **0.07** | **0.98** |
| **GLCA** | **32.15%** | **0.10** | **0.98** | **23.34%** | **0.12** | **0.96** |
| **GUDCA** | **4.66%** | **0.10** | **0.96** | **3.54%** | **0.10** | **0.99** |
| **HDCA** | **96.93%** | **NA** | **NA** | **96.35%** | **NA** | **NA** |
| **LCA** | **36.81%** | **0.52** | **0.61** | **27.21%** | **0.35** | **0.82** |
| **MCA (a)** | **100.00%** | **NA** | **NA** | **100.00%** | **NA** | **NA** |
| **MCA (b)** | **100.00%** | **NA** | **NA** | **100.00%** | **NA** | **NA** |
| **MCA(o)** | **100.00%** | **NA** | **NA** | **99.89%** | **NA** | **NA** |
| **TCA** | **35.95%** | **0.10** | **0.97** | **22.12%** | **0.09** | **1.00** |
| **TCDCA** | **2.58%** | **0.08** | **0.97** | **2.21%** | **0.08** | **0.98** |
| **TDCA** | **2.70%** | **0.08** | **0.96** | **1.66%** | **0.13** | **0.96** |
| **TLCA** | **25.89%** | **0.17** | **0.90** | **33.30%** | **0.24** | **0.63** |
| **TMCA(a+b)** | **28.22%** | **0.13** | **0.96** | **15.38%** | **0.13** | **0.96** |
| **TUDCA** | **5.52%** | **0.22** | **0.90** | **6.64%** | **0.11** | **0.95** |
| **UDCA** | **39.75%** | **0.14** | **0.96** | **7.85%** | **0.15** | **1.00** |
